# Perchloride of Iron as an Hœmostatic

**Published:** 1856-10

**Authors:** 


### PERCHLORIDE OF IRON AS AN HÆMOSTATIC.

A correspondent of the *Moniteur des Hopitaux* (No. 24, 1856), states that one of the principal elements of his success in the difficult and dangerous operations M. Maissonneuve is famous for undertaking, is the remarkable use he makes of hæmostatics during their performance. He cites a recent case, occurring in a lad of sixteen, of fungous tumor of the dura mater, the growth of which, after having been temporarily arrested by ligature of the carotid, took on enormous proportions, and was accompanied by exhausting hemorrhages. M. Maissonneuve determined upon its removal, but the tumor bled on the slightest contact, and the patient would not be able to bear the slightest loss of blood. The line of incision extended from the anterior parts of the ear to the summit of the head, and descending along the nose, was carried backwards, and then upwards to the base of the jaw, and its point of departure. A great number of arteries were thus divided, five or six of which, by reason of their anastomotic enlargements, had acquired almost the size of the radial artery. Intelligent assistants immediately compressed them with the finger, but it was impossible to thus continue the dissection without exposing the patient to the danger of death from syncope. M. Maissonneuve therefore applied to each vessel a little pledget of charpie soaked in perchloride of iron, which was allowed to attach itself to the wound. At every stroke of the bistoury or scissors he applied a new plug, so that during the operation the patient scarcely lost a spoonful of blood; and when the tumor had been entirely removed, the entire surface of the wound was found completely dried and tanned, and was at once dressed, without the necessity of the application of a single ligature. The brown eschar which covered the wound was detached about the twentieth day, without giving rise to any hemorrhage; and although the cure can scarcely be expected to prove radical, the patient, for the present, is perfectly well.—*Med. Times and Gazette*, *Aug.* 16*th*, 1856.

